# EVALUATING THE BEHAVIORAL RECOVERY OUTREACH (BRO) TEAM MODEL: TEAM MEMBERS’ PERSPECTIVES

**DOI:** 10.1093/geroni/igad104.1454

**Published:** 2023-12-21

**Authors:** Amanda Peeples, Elizabeth MacDonald, Kathleen Matthews

**Affiliations:** Corporal Michael J. Crescenz VA Medical Center, Philadelphia, Pennsylvania, United States; VHA Serious Mental Illness Treatment and Evaluation Center, Ann Arbor, Michigan, United States; VHA VISN 23., Des Moines, Iowa, United States

## Abstract

The VA’s Behavioral Recovery Outreach (BRO) Team model seeks to address the unmet needs of aging Veterans with complex behavioral, psychological, neurocognitive, and medical concerns. Consisting of a core team of a social worker, nurse, and psychologist, BRO teams collaborate with the broader VA Community Living Center or inpatient team on behavioral assessment and treatment planning to help stabilize the Veterans’ behaviors and ready them for discharge to the community. Then, upon discharge the BRO Team provides ongoing behaviorally focused transitional support services to help ensure the Veterans’ sustained stability in the community and prevent unnecessary rehospitalizations. While dissemination efforts have demonstrated positive clinical outcomes for the Veterans served, little was known about the factors that support or hinder successful implementation of this complex program. This paper presents results from qualitative interviews with ten members from seven BRO Teams (out of ten active teams at the time) undertaken in late 2021 as part of a larger BRO program evaluation. The BRO Team model, including the goals and core components of BRO, are defined. Determinants of successful BRO implementation and team operations, including staffing, inter-departmental relationships, model clarity, and support, are described. BRO Team members’ views of the impact on and response of BRO recipients (BRO-enrolled Veterans, family members, and VA and community care partners), such as helping family members understand dementia, assistance with navigating the VA system, and support with behavioral health care planning and modification leading up to and following transitions to community care, are also detailed.

